# An Unbiased Assessment of the Role of Imprinted Genes in an Intergenerational Model of Developmental Programming

**DOI:** 10.1371/journal.pgen.1002605

**Published:** 2012-04-12

**Authors:** Elizabeth J. Radford, Elvira Isganaitis, Josep Jimenez-Chillaron, Joshua Schroeder, Michael Molla, Simon Andrews, Nathalie Didier, Marika Charalambous, Kirsten McEwen, Giovanna Marazzi, David Sassoon, Mary-Elizabeth Patti, Anne C. Ferguson-Smith

**Affiliations:** 1 Department of Physiology, Development and Neuroscience, University of Cambridge, Cambridge, United Kingdom; 2 Research Division, Joslin Diabetes Center and Harvard Medical School, Boston, Massachusetts, United States of America; 3 Bioinformatics Group, The Babraham Institute, Cambridge, United Kingdom; 4 Myology Group-UMR S 787, INSERM and Université Paris VI/Pierre et Marie Curie, Paris, France; University of Pennsylvania, United States of America

## Abstract

Environmental factors during early life are critical for the later metabolic health of the individual and of future progeny. In our obesogenic environment, it is of great socioeconomic importance to investigate the mechanisms that contribute to the risk of metabolic ill health. Imprinted genes, a class of functionally mono-allelic genes critical for early growth and metabolic axis development, have been proposed to be uniquely susceptible to environmental change. Furthermore, it has also been suggested that perturbation of the epigenetic reprogramming of imprinting control regions (ICRs) may play a role in phenotypic heritability following early life insults. Alternatively, the presence of multiple layers of epigenetic regulation may in fact protect imprinted genes from such perturbation. Unbiased investigation of these alternative hypotheses requires assessment of imprinted gene expression in the context of the response of the whole transcriptome to environmental assault. We therefore analyse the role of imprinted genes in multiple tissues in two affected generations of an established murine model of the developmental origins of health and disease using microarrays and quantitative RT–PCR. We demonstrate that, despite the functional mono-allelicism of imprinted genes and their unique mechanisms of epigenetic dosage control, imprinted genes as a class are neither more susceptible nor protected from expression perturbation induced by maternal undernutrition in either the F1 or the F2 generation compared to other genes. Nor do we find any evidence that the epigenetic reprogramming of ICRs in the germline is susceptible to nutritional restriction. However, we propose that those imprinted genes that are affected may play important roles in the foetal response to undernutrition and potentially its long-term sequelae. We suggest that recently described instances of dosage regulation by relaxation of imprinting are rare and likely to be highly regulated.

## Author Summary

Environmental perturbations during early life are known to affect one's risk of metabolic disease many years later. Furthermore, that risk can be inherited by future generations, although the mechanisms responsible are poorly understood. Imprinted genes are unusual as only one of the two copies is expressed in a parent-of-origin–specific manner. As only one copy is active, imprinted gene dosage has been hypothesised to be uniquely vulnerable to environmental change. Therefore, it has been suggested that imprinted genes may play an important role in the developmental origins of health and disease. Alternatively, the opposite may be true—imprinted genes may be more tightly safeguarded from perturbation. To test these two hypotheses, we analysed the expression of imprinted genes in the context of all active genes in two affected generations of a mouse model of the developmental origins of health and disease. Our data show that imprinted genes as a class are neither more nor less susceptible to expression change, but a subset of imprinted genes may be involved in the adaptation of the conceptus. Furthermore, imprints in the developing germline are not affected and imprinted genes are largely stable in the second generation. This is important, as it is the first time that this hypothesis has been tested in an unbiased fashion.

## Introduction

Animal models in multiple species have confirmed that early life represents a critical window of phenotypic plasticity, highly responsive to maternal behaviour, stress, metabolism and nutrition (reviewed by [1]). Epigenetic mechanisms, “*the structural adaptation of chromosomal regions so as to register, signal or perpetuate altered activity states*” [2] are fundamentally involved in the specification of cellular phenotype. We and others have hypothesised that a compromised *in utero* environment may impinge upon the epigenetic apparatus with lasting consequences for gene expression and development. Changes in DNA methylation and histone modifications at putative regulatory regions correlating with the altered expression of genes implicated in phenotypic development have been observed in a number of animal models of early life compromise [3]–[8]. Such epigenetic modifications are hypothesised to contribute to the stable maintenance of phenotype long after exposure to the environmental insult.

The impact of the early life environment has been observed to extend over multiple generations in both human populations and animal models (for example [4], [9]–[11]). Several potential mechanisms of such non-Mendelian phenotypic inheritance can be considered. For example, transmission via the maternal line often, though not always, involves the recapitulation of the initial environmental trigger, as with the heritability of maternal reproductive behaviour [3]–[4], [12]. However, paternal transmission of environmentally induced phenotypes has also been documented [13]–[14], [15]–[17]. This strongly implicates intergenerational epigenetic inheritance because rodent males only contribute to the future generation through the sperm. However, which epigenetic mechanism(s) are responsible remains unknown. Transgenerational epigenetic inheritance of DNA methylation has been demonstrated through both maternal and paternal lineages at the *A^vy^* and *Axin^Fu^* murine alleles, formed by the insertion of IAP elements into or near to endogenous genes [18]–[19]. Furthermore, maternal gestational diet affects methylation at these loci in both the offspring and grand-offspring [20]. It is hypothesised that endogenous loci which have an inherent epigenetic vulnerability to environmental conditions may behave similarly to *A^vy^* and *Axin^Fu^* and may play an important role in the developmental origins of health and disease.

Imprinted genes, which are functionally mono-allelic in a parental-origin specific manner and subject to multiple layers of epigenetic control of expression, have been hypothesised to be particularly vulnerable to environmental perturbation [21]. Imprinted genes have been shown to regulate the development of key metabolic organs and have therefore been proposed as good candidates to play a role in the developmental origins of health and disease (reviewed by [22]). Furthermore, as germ cell epigenetic reprogramming of imprinting control elements occurs at least partially *in utero*, it has been postulated that deregulation of this process may be involved in phenotypic inheritance by the next generation. However, it has also been hypothesised that the converse may instead be true: given the dependence of imprinted gene dosage on multiple layers of epigenetic regulation, imprinted gene expression may be more tightly safeguarded in the face of environmental perturbations during development and any mechanism inducing the action of the canonically repressed allele would be highly regulated [23]. Proper investigation of these hypotheses requires the analysis of how the expression of imprinted genes, as a class, responds to environmental challenge relative to the whole transcriptome and compared to other functionally related gene sets.

Our aim therefore was to investigate the role of imprinted gene expression in an established murine model of developmental programming. Specifically we aimed to assess imprinted gene expression in the context of the transcriptome to test whether imprinted genes, as a class, are more or less susceptible than bi-allelically expressed genes to perturbation in expression resulting from gestational undernutrition. We have previously reported that the F1 offspring of dams subjected to 50% caloric restriction during the last week of gestation have a phenotype of low birth weight associated with early-life adiposity, altered pancreatic function and progressive glucose intolerance [24]. In this model, both paternal and maternal inheritance of glucose intolerance to the F2 generation is observed in the absence of any further environmental perturbation [15]. Candidate-based qPCR and microarrays were employed to assess the contribution of genomic imprinting to the developmental origins of health and disease in the F1 and F2 generations of this model.

## Results

### Analysis of imprinted gene expression in the context of the transcriptome of the E16.5 F1 generation

**Figure 1:**
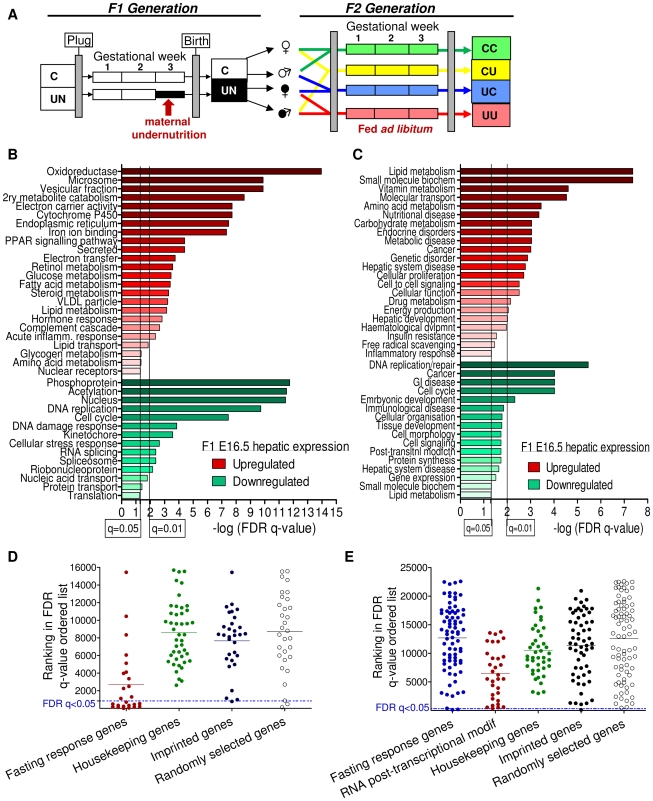
Characterisation of the E16.5 hepatic and placental transcriptome response to undernourishment. (A) Schematic of the experimental design: F1 generation: On pregnancy day 12.5, dams were randomly assigned to either control or undernutrition groups and food intake of undernutrition mothers was restricted to 50% that of controls. After delivery litter size was equalized to eight pups and dams received 9F chow *ad libitum*. Pups nursed freely and were weaned at 3 weeks onto 9F chow *ad libitum*. F2 generation: control and undernourished females from the F1 generation were mated at age 2 months with nonsibling control or undernourished males. After confirmation of pregnancy, females were caged individually and fed *ad libitum* throughout pregnancy to produce the four experimental F2 generation groups: CC – both parents are controls. CU – control dam, *in utero* undernourished sire; UC - *in utero* undernourished dam, control sire; UU - *in utero* undernourished dam, *in utero* undernourished sire. (B, C) Gene ontology (GO) analysis of the F1 undernourished hepatic transcriptome at E16.5: Functional enrichment analysis using (B) DAVID [27]–[28] and (C) Ingenuity Pathway Analysis functional analysis tools. Among genes upregulated in the liver, both tools identify significant enrichment (after Benjamini-Hochberg correction for multiple testing) of gene groups associated with metabolism, particularly of lipids. Among genes downregulated in the liver, both tools identify significant enrichment of categories related to cell-cycle and the control of proliferation. (D) Transcriptome analysis of E16.5 undernourished versus control liver. Distribution of the ranked difference in gene expression according to FDR q-value. Lists of housekeeping genes and adult hepatic fasting-response genes curated from the literature were used as negative and positive controls respectively and their rankings shown. Imprinted genes most closely resemble randomly selected genes. (E) Transcriptome analysis of E16.5 undernourished versus control placenta; distribution of genes according to FDR q-value. The placental response to maternal undernourishment is undetermined, and is different to that of the liver, as fasting response genes are largely unperturbed. Therefore a network of genes involved in RNA post-transcriptional modification, identified as enriched in undernourished placenta by IPA analysis; was used as a positive control. Imprinted genes most closely resemble randomly selected genes.

Since expression of most imprinted genes diminishes towards term and during early postnatal life (our observations, [25]), expression was assessed at E16.5, see Figure 1A. Transcriptome analysis of E16.5 liver of control (C) and *in utero* undernourished (UN) F1 animals demonstrated 765 genes with significantly perturbed expression in UN liver (false discovery rate, FDR, q<0.05 following Benjamini-Hochberg correction for multiple testing). Of the affected genes, 383 were up-regulated and 382 downregulated. Power to resolve a 1.5 fold change in expression was estimated to be 99%. In E16.5 placenta, 304 genes were significantly affected in UN conceptuses (FDR q<0.05). Of the affected genes, 170 were up-regulated and 134 downregulated. Power to resolve a 1.5 fold change in expression was estimated to be 75%. Over 70% of all imprinted genes are represented on these arrays. Of the imprinted genes on the array, the majority were found to be expressed in F1 E16.5 placenta and liver (78% and 54% respectively).

A single imprinted gene, *Grb10*, was identified as being significantly affected in E16.5 F1 UN liver. Similarly, *IMPACT* was the only imprinted gene affected in E16.5 UN placenta. Although power was estimated to be relatively high, imprinted genes are rare and it is conceivable that modest changes in expression of multiple imprinted genes may play a significant role in F1 phenotype but would fall beneath the multiple-testing correction threshold for significantly altered gene expression. Gene Set Enrichment Analysis, GSEA, can be employed to investigate the expression of *a priori* defined gene sets [26]. GSEA did not identify significant enrichment of the imprinted gene set in either the hepatic or placental transcriptional profile of C or UN samples (normalised enrichment score (NES) 1.31 and FDR 0.81 in UN liver; NES 0.84 and FDR 0.88 in UN placenta). In agreement with this, there was no difference in the ROC area under the curve between imprinted and randomly permuted gene sets for F1 E16.5 liver and array data (Figure S1A). This suggests that imprinted genes as a class are not particularly susceptible to expression perturbation following *in utero* undernutrition.

This analysis could be confounded if the foetal transcriptional response to starvation was not fully developed by E16.5, four days into maternal undernutrition, or if cellular heterogeneity and/or inter-individual variation meant that the experimental design did not have sufficient power to detect biologically relevant changes in gene expression. The adult transcriptional response to starvation promotes a switch from glucose to fatty acid and ketone body utilisation. Gene ontology analysis of the F1 hepatic transcriptome data using both DAVID [27]–[28] and Ingenuity Pathway Analysis tools demonstrated enrichment of terms relating to metabolic function among the genes up-regulated in undernourished foetal liver; these included “PPAR signalling pathway”, “Lipid metabolism”, “Fatty acid metabolism”, “Lipid transport”, “The role of Nuclear Receptors in lipid metabolism” (Figure 1B, 1C). Gene set enrichment analysis corroborated these data: gene sets associated with regulation of the key metabolic transcription factors FXR and LXR, “Lipid catabolic process”, “Fatty acid oxidation”, “Lipid transport”, “Starch and sucrose metabolism” and “Oxidoreductase activity” were enriched in undernourished F1 liver (Table 1). This suggests that the hepatic metabolic transcriptional response to maternal nutritional deprivation is well developed at E16.5, and validates E16.5 as an appropriate time point to assess the role of imprinted genes in this process. These data also demonstrate that biologically relevant changes in gene expression patterns are successfully distinguished from the background “noise”, and that the foetal hepatic response to starvation involves an upregulation of lipid catabolism, similar to that of the adult.

**Table 1:** Gene set enrichment analysis of E16.5 F1 hepatic transcriptome response to undernutrition.

Gene set	Enriched in	Normalised enrichment score	q-value
Imprinted genes	UN	1.31	0.81
FXR regulated in muscle	UN	2.44	0.000
Lipid catabolic process	UN	2.29	0.000
LXR regulated in muscle	UN	2.27	0.000
Fatty acid oxidation	UN	2.19	0.000
Lipid transport	UN	2.11	0.000
Starch and sucrose metabolism	UN	1.94	0.006
Oxidoreductase activity	UN	1.83	0.0018
Response to oxidative stress	UN	1.63	0.065
Insulin receptor signalling pathway	UN	1.60	0.066
Cell cycle	C	2.4	0.000
Caspase pathway	C	2.21	0.001
P27 pathway	C	2.17	0.000
Cell cycle checkpoint	C	2.01	0.001
mRNA splicing	C	1.93	0.002
TNFR1 pathway	C	1.84	0.005

To compare the response to *in utero* undernourishment of imprinted genes versus that of other, functionally related gene sets, all genes on the array were ordered according to their FDR q-value of expression change. The distribution of imprinted genes in this FDR ordered list was plotted and compared to a randomly selected group of genes; a group of housekeeping genes expected to be protected from expression change, and a positive control of genes expected to have altered expression in undernourished tissues. As a positive control of a group of genes expected to have altered expression in F1 undernourished foetal liver, a gene set was curated from the literature documenting the adult hepatic transcriptional response to starvation [29]–[32]. As no comparable studies have been done in placenta, a group of genes identified as enriched by gene ontology analysis using the IPA tool, genes involved in the post-transcriptional modification of RNA, were used as a positive control.

If imprinted genes, as a class, are more susceptible to *in utero* undernourishment, they would be expected to resemble those genes involved in the starvation response. Conversely, if imprinted genes, as a class, are protected from environment-induced transcriptional perturbation, they would be expected to resemble housekeeping genes. However, imprinted genes most closely resemble the randomly selected gene group in both liver and placenta (Figure 1C, 1D). This suggests that imprinted genes are neither more susceptible to, nor protected from transcriptional perturbation induced by *in utero* nutrient restriction in the F1 generation.

### Altered expression of a subset of imprinted genes may play an important role in the phenotypic development of the F1 generation

**Figure 2:**
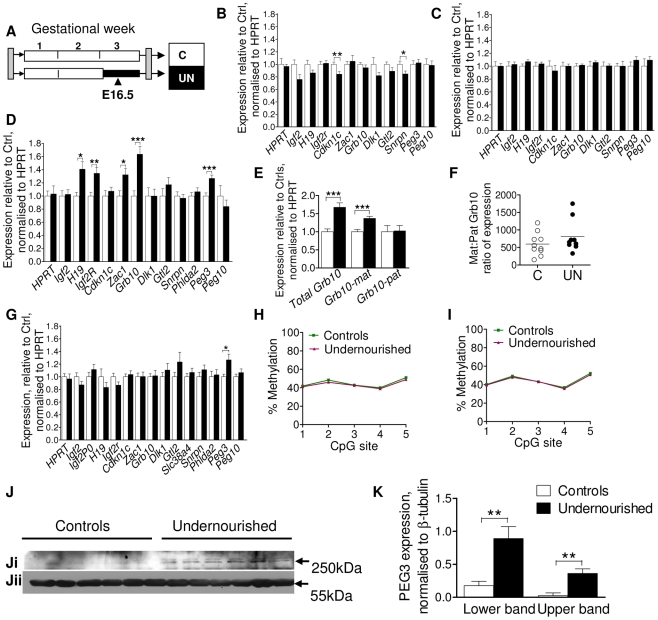
Assessment of candidate imprinted genes at E16.5 in the F1 generation. (A) Schematic of the F1 generation, imprinted gene expression was assessed at E16.5, the mid-point of maternal caloric restriction. (B–G) Imprinted gene expression assayed by qPCR, normalised to *HPRT* and expressed relative to controls: Open bars/circles represent controls, black bars/circles, undernourished individuals. Error bars show SEM. (B) Brain. Per condition n = 12, 3 litters. Unpaired two-tailed t-test *Cdkn1c* P = 0.009, *Snrpn* P = 0.036. (C) Muscle (tongue): per condition n = 12, 3 litters. (D) Liver: per condition n = 36, 5 litters *H19* P = 0.02, *Igf2r* P = 0.008, *Zac1* P = 0.02, *Grb10* P = 0.0002, *Peg3* P = 0.005. (E) The increased hepatic expression of Grb10 in F1 E16.5 liver is of the appropriate maternal-type isoforms (*mGrb10α* and *mGrb10δ*). Per condition n = 36, 5 litters *Grb10* P = 0.0002; *matGrb10* P<0.0001. (F) In the few individuals where some expression of paternal-type isoforms (*mGrb10β1* and *mGrb10β2*) is detectable in E16.5 liver, this expression is dwarfed by that of maternal-type isoforms. (G) Placenta: per condition n = 24, 5 litters. *Peg3* P = 0.017. (H, I) Methylation at the Peg3 promoter DMR assessed by pyrosequencing. Data presented are the average of three independent bisulphite treatments. Per condition n = 12, 3 litters, error bars show SEM. *In utero* undernutrition does not affect the methylation status of the Peg3 promoter DMR in (H) F1 E16.5 brain (I) F1 E16.5 liver. (J) Western blot of PEG3 (Ji) and β-tubulin (Jii) using brain tissue of E16.5 control and undernourished foetuses. Per condition n = 6, 3 litters. (K) Although *Peg3* mRNA is unaffected (Figure 3B), expression of PEG3 is increased relative to β-tubulin in the brain of undernourished E16.5 foetuses. Data represent the average of two technical replicates. Error bars denote SEM.

In order to expand the number of individuals assessed and the number of tissues interrogated, expression of a group of candidate imprinted genes was measured by quantitative PCR in four tissues: liver, muscle, placenta and brain at E16.5, according to tissue-specific imprinting patterns (Figure 2B–2G). Altered dosage of these imprinted genes has previously been shown to perturb foetal and placental growth and affect the development of metabolic organs and axes, with long-term implications for adult metabolism (reviewed in [22]). These tissues were chosen as their development has been shown to be sensitive to early life conditions, susceptible to imprinted gene dosage, and to be critical to metabolic health. In brain, muscle and placenta, imprinted gene expression at E16.5 is largely stable, although brain expression of *Cdkn1c* and *Snrpn* is significantly but subtly reduced in undernourished animals, and placental expression of *Peg3* (also called *Pw1*) is increased as shown in Figure 2B, 2C, 2G. However, in E16.5 liver *H19*, *Igf2r*, *Zac1*, *Peg3* and *Grb10* are significantly up-regulated by maternal undernutrition (Figure 2D). *Grb10* is expressed from the maternally-inherited allele in the liver, but from the paternally-inherited allele in the brain, and these two transcript classes utilise alternative transcriptional start sites [33]. The increase in *Grb10* expression in F1 UN E16.5 liver is of the usually expressed maternal-type isoforms (Figure 2E, 2F).

Sexual dimorphism is frequently observed in animal models of developmental programming [16], [34]–[35]. In this model both sexes are affected, although males more frequently than females [24]. While sex had no impact on expression of the majority of imprinted genes, hepatic *Igf2r* and *Zac1* were significantly upregulated only in undernourished females (*Igf2r* UN females average fold change (FC) = 1.82, unpaired two-tailed t-test P = 0.0008 (95% CI 1.35–2.29), UN males FC = 1.13 (95% CI 0.98–1.28); *Zac1*: UN females FC = 1.88, P = 0.001 (95% CI 1.36–2.39), UN males FC = 1.15 (95% CI 0.85–1.45)). In the placenta, the undernutrition induced upregulation of *Peg3* was male specific (UN males FC = 1.50, P = 0.02 (95% CI 1.13–1.86), UN females FC = 1.04 (95% CI 0.85–1.22)). Hepatic upregulation of *Peg3* occurred in both sexes, demonstrating that sex-dependent effects are tissue-specific. While sex-specific effects may increase the level of background expression variation, our data suggest we have sufficient power to detect them, even when analysing both sexes together.

Altered imprinted gene expression can be brought about by canonical transcription-factor mediated mechanisms, or through the loss or relaxation of imprinting. Loss of imprinting results in either the silencing of the normally expressed allele or the activation of the normally silenced allele and is associated with alterations in the epigenetic marks which control allele-specific expression. Increased *Peg3* expression was observed in liver and placenta, but not brain or muscle. To assess whether this was the result of tissue-specific changes in imprinting control, we quantified the level of methylation at the *Peg3* promoter, a maternally methylated germline differentially methylated region, DMR, by pyrosequencing in brain and liver. *Peg3* DMR methylation was at the expected level of ∼50% in control individuals (Figure 2H, 2I) and was unchanged in both brain and liver in UN individuals. As we are assaying a heterogenous cell population, it is theoretically possible that a very small subset of cells may have more substantially perturbed methylation. We also cannot assess the relative distribution of methylation between the maternally and paternally inherited alleles, and pyrosequencing cannot readily distinguish between cytosine methylation and hydroxymethylation. However, overall these data suggest that the modulation in *Peg3* expression is likely to be through the transcription-factor mediated upregulation of the canonical paternally expressed allele, and not due to a relaxation of imprinting.

*Peg3* has been implicated in the central regulation of energy balance, and is known to affect maternal gestational nutrient partitioning, in addition to playing a role in reproductive and nurturing behaviour [36]–[37]. PEG3 is highly expressed in the foetal and the adult hypothalamus [36], [38]–[39], and alterations in central PEG3 dosage in the F1 generation may have implications for the phenotypic development of the F2 generation. Although no change in brain *Peg3* expression was observed at the mRNA level, a clear induction of PEG3 was observed by Western blot in the brains of UN animals (Figure 2J, 2K).

### F2 generation

The metabolic phenotype observed in the F1 generation is transmitted to the F2 generation in the absence of further dietary compromise [15], [40]. The reliance of imprinting control on parental-origin specific differential epigenetic marks necessitates the erasure and sex-specific reapplication of these marks during germ cell development (reviewed in [41]). The *de novo* methylation of imprinting control regions occurs asynchronously in the male and female gametes [42]–[45]. Consequently, maternal nutritional restriction during the third gestational week coincides with the re-acquisition of methylation at imprinting control regions in the primordial germ cells of the male but not the female F1 embryo [42]–[45]. Recent data have suggested that the maternally-methylated primary DMRs are distinguishable as unmethylated islands in sperm, raising the possibility that they are protected from *de novo* methylation in the paternal gametes [46]. Therefore, we hypothesised that if imprinted genes are susceptible to methylation change in primordial germ cells due to *in utero* nutritional restriction, this will be most evident in the offspring of F1 generation males. To test this hypothesis we assessed placental and hepatic imprinted gene expression at E16.5 in the F2 generation and directly analysed sperm methylation in F1 males.

**Figure 3:**
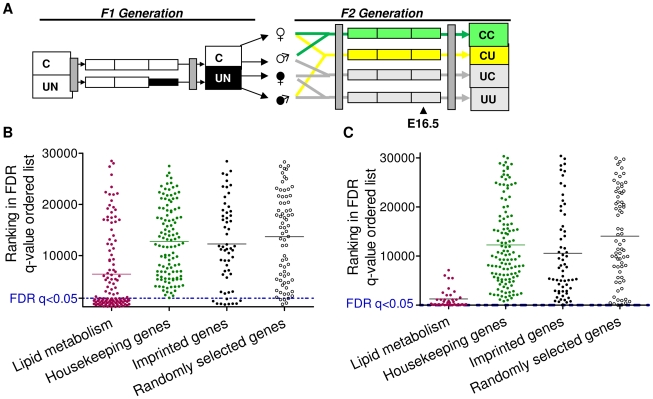
Characterisation of the F2 CU hepatic and placental transcriptome. (A) Schematic of the F2 generation, the transcriptome of the CU and CC F2 crosses was assessed at E16.5. (B) Distribution of the ranked difference in gene expression in CU E16.5 liver according to FDR q-value. A list of genes comprising a network involved in lipid metabolism, identified as enriched in CU liver using IPA software was used as a positive control. Imprinted genes most closely resemble randomly selected genes. (C) Distribution of genes differentially expressed in CU E16.5 placenta according to FDR q-value. A list of genes comprising a network involved in lipid metabolism, identified as enriched in CU placenta using IPA software was used as a positive control. Imprinted genes most closely resemble randomly selected genes.

To assess the impact of paternal *in utero* undernutrition on the expression of imprinted genes in the context of the whole transcriptome, expression was assessed by microarray in the E16.5 liver and placenta of F2 foetuses with a control dam and an *in utero* undernourished sire (CU), and compared to foetuses whose parents had never experienced *in utero* undernourishment (CC) (Figure 3A).

In E16.5 F2 liver, 1330 genes demonstrated significantly different expression levels between CC and CU animals (FDR q<0.05 following BH correction for multiple testing). Of the affected genes, nearly three quarters (72%) were downregulated. Power to resolve a 1.5 fold change was estimated to be 87%. In E16.5 F2 placenta, only 4 genes demonstrated significantly different expression levels between controls and CU placentas (FDR q<0.05). However, power to resolve a 1.5 fold change was estimated to be 64%. The placenta has a greater variety of cell types than the liver and is morphologically plastic in response to foetal and maternal cues [47]. This may have resulted in a greater inter-individual variability of gene expression which may account for the reduced statistical power. Over 78% of all imprinted genes are assayed by these arrays. Of the imprinted genes on the array, the majority were found to be expressed in F2 E16.5 placenta and liver (94% and 86% respectively).

Gene ontology analysis did not detect genomic imprinting as significantly enriched in either liver or placenta (genes with altered expression >1.5 fold were used for GO analysis in the F2 placental data set). ROC curve analysis in F2 CU liver suggested that imprinted genes are moderately more likely to have a lower FDR than non-imprinted gene sets (area under the curve was higher in the imprinted gene set compared to 99/100 randomly permuted gene sets), see Figure S1A. However, GSEA did not detect any statistically significant enrichment of imprinted genes among the altered expression profile in either E16.5 liver or placenta (NES 1.28, FDR 1.00; NES 1.00, FDR 0.84 for enrichment in CU group in liver and placenta, respectively). This suggests that imprinted genes, as a class, are not particularly vulnerable to expression perturbation in the offspring of *in utero* undernourished males, and suggests that the re-programming of imprinting control elements in F1 primordial germ cells is unaffected by caloric restriction.

As described above for the F1 generation, we compared the distribution of imprinted genes in a FDR ordered list to a randomly selected group of genes, housekeeping genes hypothesised to be protected from changes in expression and gene sets found to be enriched in F2 CU liver and placenta by gene ontology analysis (IPA). The distribution of imprinted genes in both the F2 E16.5 hepatic and placental transcriptomes most closely resembles that of the randomly selected gene group (Figure 3B, 3C). These data indicate that, as with the F1 generation, imprinted genes are neither more susceptible, nor protected from changes in gene expression in E16.5 liver or placenta.

### qPCR analysis

**Figure 4:**
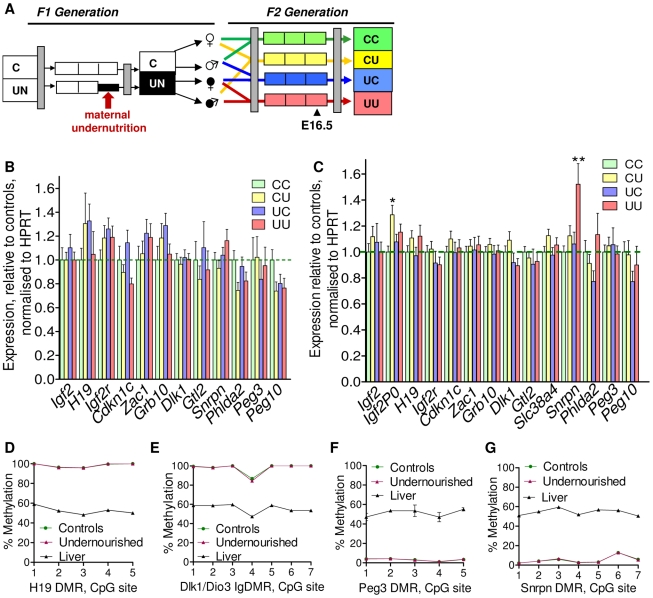
Expression of candidate imprinted genes at E16.5 in the F2 generation and assessment of F1 germline imprints. (A) Schematic of the F2 generation, expression was assessed at E16.5. (B) There are no significant differences in the F2 hepatic expression of imprinted genes at E16.5. Error bars denote SEM. (C) At E16.5 CU individuals demonstrate a significant increased in placental expression of *Igf2P0* (One-way ANOVA, Bonferroni's multiple comparison post-test. P<0.05), while UU placentas significant up-regulate *Snrpn* (One-way ANOVA, Bonferroni's multiple comparison post-test. P<0.01). Per condition n≥24, 6 litters. Error bars denote SEM. (D, E) Methylation was assessed by pyrosequencing at the paternally methylated *H19* (A) and *Dlk1/Dio3* (B) germline ICRs. Sperm from both control and *in utero* undernourished males showed the expected hypermethylation in comparison to somatic tissues (liver). Controls: n = 12, 5 litters. Undernourished n = 11, 4 litters. Error bars denote SEM. (F, G) Pyrosequencing assessment of methylation at the maternally methylated *Peg3* (C) and *Snrpn* (D) germline DMRs shows that these regions are unmethylated in the sperm of both control and *in utero* undernourished males. Controls: n = 12, 5 litters Undernourished n = 11, 4 litters. Error bars denote SEM.

As a preliminary assessment of the role of imprinted genes in all the F2 crosses, expression of a group of candidate metabolically important imprinted genes was measured by quantitative PCR in liver and placenta at E16.5, see Figure 4B. While there is some variability in the hepatic expression of certain imprinted genes in the F2 generation at E16.5, particularly *Igf2r*, *Grb10*, *Zac1* and *Cdkn1c*, differences between groups do not reach statistical significance, see Figure 4B (one-way ANOVA, Bonferroni's multiple comparison test). Thus, we can conclude that imprinted gene expression is not significantly affected in the F2 E16.5 liver. Despite the increased cellular heterogeneity of this tissue, expression of imprinted genes in F2 E16.5 placenta is generally less variable than that of F2 E16.5 liver. Expression of *Igf2P0* is significantly increased in CU placentas and *Snrpn* in UU placentas, as shown in Figure 4C (one-way ANOVA, Bonferroni's multiple comparison test). There was no sexual dimorphism in expression changes of imprinted genes in F2 tissues (data not shown).

To quantify the variation in expression attributable to the *in utero* nutrition of each parent, and to ascertain whether there is any interaction between these two variables these data were re-analysed using a two-way ANOVA. These results, presented in Table 2 and Table 3, demonstrate that parental *in utero* undernutrition does not have a significant impact on the expression of the majority of imprinted genes at E16.5 in liver and placenta. Expression of five out of the fourteen genes tested in the placenta had a significant component of variation attributable to parental *in utero* nutrition. *Igf2P0* was significantly affected by both maternal and paternal nutrition, while for *Snrpn* and *H19* only paternal nutrition and for *Dlk1* only maternal nutrition contributed significantly to expression variation, while there was a significant interaction between maternal and paternal nutrition on *Phlda2* expression. In F2 E16.5 liver, expression of three out of the twelve genes tested demonstrated a significant component of variation attributable to parental nutrition – paternal nutrition on *Cdkn1c* and *Phlda2* expression and maternal nutrition on *Snrpn* expression. Across both liver and placenta there was no discernable relationship between which parent's nutrition had a significant effect and whether the gene was expressed from the paternally inherited or maternally inherited allele.

**Table 2:** Analysis of F2 hepatic candidate imprinted gene expression by two-way ANOVA.

Gene	Contribution to total expression variation (%):		
	Maternal *in utero* nutrition	Paternal *in utero* nutrition	Interaction
*Igf2*	0.20 (ns)	0.27 (ns)	0.19 (ns)
*H19*	1.34 (ns)	0.64 (ns)	1.68 (ns)
*Igf2r*	1.31 (ns)	0.76 (ns)	2.63 (ns)
*Cdkn1c*	0.07 (ns)	5.85 * (P = 0.014)	1.67 (ns)
*Zac1*	2.46 (ns)	0.01 (ns)	0.14 (ns)
*Grb10*	0.37 (ns)	0.05 (ns)	2.8 (P = 0.051)
*Dlk1*	0.29 (ns)	0.18 (ns)	0.04 (ns)
*Gtl2*	0.31 (ns)	1.14 (ns)	0.01 (ns)
*Snrpn*	7.73 ** ( P = 0.0043)	0.53 (ns)	2.26(ns)
*Phlda2*	0.01 (ns)	5.31 * (P = 0.02)	0.66 (ns)
*Peg3*	6.00 (ns)	0.03 (ns)	0.40 (ns)
*Peg10*	2.24 (ns)	3.01 (ns)	1.98 (ns)

**Table 3:** Analysis of F2 placental candidate imprinted gene expression by two-way ANOVA.

Gene	Contribution to total expression variation (%) of:		
	Maternal *in utero* nutrition	Paternal *in utero* nutrition	Interaction
*Igf2*	0.05 (ns)	0.06 (ns)	1.02 (ns)
*Igf2P0*	3.59 * (P = 0.021)	5.83 ** (P = 0.004)	1.91 (ns)
*H19*	0.02 (ns)	4.00 * (P = 0.045)	0.11 (ns)
*Igf2r*	2.42 (ns)	0.00 (ns)	0.07 (ns)
*Cdkn1c*	0.35 (ns)	1.21 (ns)	0.26 (ns)
*Zac1*	0.04 (ns)	0.42 (ns)	0.00 (ns)
*Grb10*	0.43 (ns)	0.69 (ns)	0.11 (ns)
*Dlk1*	4.98 * (P = 0.024)	0.28 (ns)	0.87 (ns)
*Gtl2*	0.71 (ns)	0.03 (ns)	0.22 (ns)
*Slc38a4*	0.58 (ns)	2.62 (P = 0.058)	0.15 (ns)
*Snrpn*	0.00 (ns)	5.79 ** (P = 0.005)	1.74 (ns)
*Phlda2*	0.17 (ns)	1.8 (ns)	3.92 * (P = 0.041)
*Peg3*	0.00 (ns)	0.02 (ns)	0.54 (ns)
*Peg10*	2.09 (ns)	0.24 (ns)	0.49 (ns)

### Sperm methylation

It is conceivable that changes in the epigenetic status of imprinted genes in the F1 sperm may result in changes in expression earlier in gestation that are not detected at E16.5, or be erased during pre-implantation methylation re-programming. Consequently, the methylation status of four germline DMRs was quantitatively assessed in F1 sperm by pyrosequencing. No changes in F1 sperm methylation profile were identified in males that had been undernourished *in utero*. The intergenic germline DMR (IG-DMR) of the *Dlk1/Dio3* locus and the *H19* DMR are paternally methylated ICRs and are hypermethylated in the sperm of both control and *in utero* undernourished F1 males (Figure 4D, 4E). In contrast, the *Peg3* and *Snrpn* DMRs are normally methylated on the maternally inherited allele in somatic tissues, and are entirely unmethylated in the sperm of both control and *in utero* undernourished F1 males (Figure 4F, 4G).

## Discussion

We analyse the role of imprinted genes in multiple tissues in two affected generations of a murine model of the developmental origins of health and disease. We demonstrate that despite the functional mono-allelicism of imprinted genes, and the multiple layers of epigenetic regulation of expression, imprinted genes as a class are neither more susceptible nor protected from hepatic or placental expression perturbation induced by maternal undernutrition in either the F1 or the F2 generation. However, our candidate based qPCR analysis demonstrates that a small subset of imprinted genes is affected in F1 tissues, while their expression is largely more stable in the F2 generation. We propose that those imprinted genes which are affected may play important roles in the foetal response to undernutrition, and potentially also the subsequent long-term sequelae. We suggest that such instances of altered expression are transcription factor mediated, although we cannot directly tested the allele specificity of expression in this model. Consistent with this, upregulation of F1 hepatic *H19* is independent of any change in *Igf2* expression, suggesting that imprinting at this cluster is not affected, while upregulation of *Grb10* is of the maternal type isoform [33]. In the case of *Peg3* in the F1 generation we find no evidence of altered methylation at the imprinting control region which would be hypothesised to accompany a loss or relaxation of imprinting. We propose that instances of dosage regulation through absence or relaxation of imprinting, such as that recently described by our laboratory [48], are selective, rare and likely to be tightly regulated.

Of the four tissues analysed in the F1 generation, the liver showed the greatest perturbation of imprinted gene expression, with upregulation of *H19*, *Igf2r*, *Zac1*, *Grb10* and *Peg3* expression. We propose that this is an important element of the foetal adaptive response to undernutrition. Interestingly, the upregulation of *Igf2r* and *Zac1* occurs only in undernourished females. IGF2R is a negative regulator of IGF2 and foetal growth is known to be sensitive to its dosage [49]–[50]. Upregulation of IGF2R would be expected to increase IGF2 turnover and reduce its anabolic actions, and may contribute to the maintenance of foetal growth within the boundaries of nutrient availability. ZAC1 has been proposed to co-ordinately modulate several imprinted pathways controlling foetal growth and development [51]. However, in these F1 livers, only a subset of the genes proposed to be downstream of ZAC1 are significantly affected, indicating that if ZAC1 is involved in the coordination of the F1 transcriptional response, this role is mitigated by other factors. As females are less frequently affected by metabolic sequelae later in life, we hypothesise that increased *Igf2r* and *Zac1* may confer some protection. However, further investigation of these preliminary data is required.

F1 hepatic expression of *Grb10*, *H19* and *Peg3* is significantly increased in both sexes following undernourishment. GRB10 has been shown *in vivo* to be a negative regulator of insulin action and foetal growth, acting downstream of the insulin and IGF pathways [52], and downstream of mTORC1, a critical nutrient and hormone-sensitive regulator of cellular growth and proliferation [53]–[54]. Consequently, upregulation of *Grb10* as part of the starvation response would be expected to suppress the hepatic response to circulating insulin and IGF2, potentially preserving blood glucose for the development of cardinal organs. The full role of *H19* has not yet been delineated, but it is thought to function as a tumour-suppressor, [55] and may negatively regulate cellular proliferation. Induction of hepatic *H19* expression in this context may contribute to the maintenance of F1 foetal growth within the boundaries of nutritional availability. We suggest that the upregulation of hepatic *Igf2r*, *Grb10*, and *H19* may contribute to the growth restriction observed at birth in the F1 generation.

We detect an increase in *Peg3* expression in F1 undernourished liver and placenta and protein levels were found to be increased in the brain of F1 undernourished animals. Although not yet fully elucidated, PEG3 has been proposed to function downstream of p53 in the regulation of cellular proliferation and apoptosis in response to environmental stressors [56]–[59]. Recent work has suggested that the adverse consequences of developmental programming may reflect accelerated ageing [8] and the p53 pathway has been shown to regulate ageing and longevity [60]–[61]. Therefore, altered dosage of PEG3 may be involved in the suppression of cellular proliferation and embryonic growth, potentially with long term consequences. Foetal PEG3 expression has also been shown to contribute towards the maternal drive for gestational energy acquisition [37], although how this occurs is uncertain. Therefore, we hypothesise that upregulation of *Peg3* by the undernourished conceptus may be an adaptive response to stimulate maternal nutritional intake. Furthermore, as PEG3 dosage affects hypothalamic development, and Peg3^+/−^ animals demonstrate defects in maternal behaviour and gestational energy partitioning, increased PEG3 expression in the developing F1 brain may have implications for the phenotype of the F2 generation.

We also present the first transcriptional characterisation of the foetal hepatic response to caloric restriction. Our data suggest that the hepatic transcriptional response of the E16.5 foetus to fasting is very similar to that of the adult. Gene ontology and gene set enrichment analysis demonstrate the substantial enrichment of gene categories relating to lipid metabolism among upregulated genes, including PPAR signalling and fatty acid metabolism (Figure 1B, 1C and Table 1). Together, these data are suggestive of an appropriate switch in foetal metabolism away from the utilisation of glucose and towards fatty acid oxidation under conditions of limited glucose availability.

In the F2 generation there is less perturbation of imprinted gene expression than in the F1 generation. For a minority of imprinted genes parental nutrition contributes a significant proportion of the observed variation in expression. However, where trends in expression are observed in CU and UC animals, with undernourished father and mother respectively, these trends are generally not additive in animals with two undernourished parents, UU animals. For example, *Igf2P0* expression is upregulated in CU placenta, but unaffected in the placenta of UU individuals. Conversely, *Snrpn* expression is increased in the placenta of UU individuals but not perturbed in either CU or UC individuals. This suggests that alteration in imprinted gene expression is part of the foetal plastic response to the *in utero* environment, and is not due to germline derived changes in epigenetic marks. Consistent with this, we find that the epigenetic re-programming of imprinting control regions in the germline, at least in males, is resistant to 50% caloric restriction. The increased expression of *Igf2P0* in the placentas of CU individuals is interesting as *Igf2P0* has been proposed to play a key role in matching placental solute transport to foetal demand [62]–[63], and may represent an adaptive placental response to support CU foetal growth. Little is currently known of the role of *Snrpn* in the placenta, but these data suggest it deserves further attention.

### Concluding remarks

In conclusion, we have demonstrated that, at least in this murine model of prenatal undernutrition, the functional mono-allelicism of imprinted genes and their unique mechanisms of epigenetic control of expression do not render them either more or less susceptible to expression perturbation following environmental challenge. Nor is there any evidence that germline reprogramming of ICRs is susceptible to nutritional restriction. However, we propose that the selective dosage modulation of certain imprinted genes plays an important role in the adaptive foetal response to *in utero* nutritional scarcity.

## Methods

### Animal protocols

ICR mice, an outbred strain, were obtained from the Jackson Laboratory. Mice were housed in an OLAW-approved facility, with controlled temperature, humidity, and light-dark cycle (07:00–19:00). Protocols were approved by the Joslin Diabetes Centre Institutional Animal Care and Use Committee. “Principles of Laboratory Animal Care” (http://grants1.nih.gov/grants/olaw/references/phspol.htm) were followed.

F1 generation (as described by [24]): Virgin female ICR mice (age 6–8 weeks) were caged with ICR male mice. Pregnancy was dated with vaginal plugs (day 0.5), and pregnant female mice were housed individually with *ad libitum* access to Purina 9F (9% fat) chow. On pregnancy day 12.5, female mice were randomly assigned to either control or undernutrition groups; weight did not differ between control and undernutrition mothers prior to pregnancy or at day 12.5. Food intake of undernutrition mothers was restricted to 50% that of controls, calculated each day, from days 12.5 to 18.5. After delivery, litter size in both groups was equalized to eight pups per dam by removing both the heaviest and lightest mice in the litter, thus retaining those with birth weight closest to the median. Mothers received chow *ad libitum* after delivery. Pups nursed freely and were weaned at 3 weeks onto 9F chow *ad libitum*.

F2 generation (as described by [15]): control and undernourished females from the F1 generation were mated at age 2 months with nonsibling control or undernourished males. After confirmation of pregnancy, females were caged individually and fed *ad libitum* with no dietary manipulation during pregnancy to produce a second, F2, generation with four experimental groups: CC – both parents are controls. CU – control dam, *in utero* undernourished sire; UC - *in utero* undernourished dam, control sire; UU - *in utero* undernourished dam, *in utero* undernourished sire. In this study, gene expression was assessed in both the F1 and F2 generations at E16.5.

### Tissue dissection

Mice were anaesthetised with intraperitoneal pentobarbital following an overnight fast and tissues were rapidly dissected and snap frozen in liquid nitrogen. To facilitate multiple extractions of DNA, RNA and protein from the same tissue, samples were pulverised in liquid nitrogen and were never allowed to thaw.

All kits were utilised according to the manufacturers' instructions, except as noted below:

### RNA extraction

RNA was extracted using Trizol (Invitrogen), with an overnight precipitation step at −20°C. Newly isolated RNA was quantified by spectrophotometric analysis and the quality was assessed by judging the integrity of the 28S and 18S ribosomal RNA bands by electrophoresis through a 1% agarose gel. All samples were treated to remove DNA contamination with DNase (using the RNase-free DNase kit, Qiagen), followed by re-precipitation.

### Quantification of gene expression

cDNA was generated from 1 µg total RNA per sample using the RevertAid H Minus cDNA synthesis kit (Fermentas) with random primers. cDNA samples were diluted 1∶20 and a six-point standard curve of two-fold dilutions was prepared from pooled cDNA. The samples and standard curve were aliquoted and stored at −80°C prior to use.

Real-time quantitative PCR with SYBR Green was performed with SensiMix (Quantace) using primers in Table S1 [33], [64]. Primers were designed to assay all annotated splice-variants of a gene where possible and were checked for specificity using NCBI nucleotide BLAST and gel electrophoresis of the PCR product. A standard curve made up of doubling dilutions of pooled cDNA from the samples being assessed was run on each plate, and quantification was performed relative to the standard curve. Target gene expression was normalised to the expression of *HPRT*, the expression of which did not differ between the groups. All primers amplified with an estimated efficiency of between 110% and 80% and there was no evidence of inhibitors present in the reaction. Reactions were carried out on a DNA engine Opticon 2 thermocycler (MJ Research).

### Microarrays

Liver gene expression was analysed in F1 E16.5 mice using Illumina Mouse WG6v2 microarrays. Samples were prepared in three pools per condition, representing a total of fifteen individuals from five independent litters per condition with the sex ratio controlled between conditions. For F1 placental arrays RNA from seven control and eight undernourished samples from independent litters was hybridised to Affymetrix MOE430A arrays. For F2 liver and placental arrays, cRNA from fifteen control conceptuses from three independent litters, and eighteen CU conceptuses from three independent litters, was hybridised to Affymetrix MOE430-2 arrays in five and six pools per condition, respectively.

Analysis of microarray data was carried out inside the R-statistical environment (http://www.r-project.org). Illumina arrays were analysed using the Lumi and Limma Bioconductor packages (www.bioconductor.org). Probes not expressed on any arrays were removed from the analysis. Variance-stabilising transformation (Lin et al., 2008) and loess normalisation was employed. Affymetrix arrays were analysed using the Affy and Limma Bioconductor packages. RMA transformation and normalisation was carried out. For all arrays probes not expressed on any arrays were removed from the analysis, the quality of normalisation was assessed by density plots of intensity and box plots of amplitude. The Benjamini-Hochberg multiple testing correction was applied to control for false discovery rate, FDR, following pairwise comparison. Genes with an FDR q-value of <0.05 were considered to be significantly differentially expressed. Power was estimated using the Bioconductor Sizepower package. The distribution of differential expression in terms of FDR for the four arrays is presented in Figure S1B. Gene ontology analysis was carried out using DAVID: Database for Analysis, Visualisation and Integrated Discovery [27]–[28] and Ingenuity Pathway Analysis, IPA, software (Ingenuity Systems www.ingenuity.com). ROC curves were computed for the imprinted gene set for each array (Figure S1A). In order to provide a context for the 4 experimentally-determined (imprinted) ROCs, we also generated ROCs from 100 randomized data sets. The randomized data sets were generated by permuting the gene labels with regard to which genes are imprinted and which are not imprinted. We calculated the area under each of these curves (AUC) and compared each of the 4 experimental data sets to the 100 randomized data sets in terms of this area.

### Generation of gene sets affected and protected from expression change

As a negative control for genes that are expected to be protected from expression change, a list of non-metabolic putative housekeeping genes was curated from the literature [65]. Imprinted genes were limited to those that have been experimentally validated (Figure S2).

F1 generation: As a positive control of a group of genes expected to have altered expression in undernourished foetal liver, a gene set was curated from the literature documenting the adult hepatic transcriptional response to starvation [29]–[32]. As no comparable studies have been done in placenta, a group of genes identified as enriched by gene ontology analysis using the IPA tool, genes involved in the post-transcriptional modification of RNA, were used as a positive control.

F2 generation: As no comparable studies exist in the literature, gene groups involved in lipid metabolism which were identified as enriched in CU liver and placenta by gene ontology analysis (Ingenuity) were used as a positive control.

### Western blot

Western blot was carried out essentially as previously described [66]. Briefly, pulverised snap frozen samples were homogenised on ice in RIPA buffer supplemented with Complete protease inhibitors (Boeringher), activated sodium orthovanadate and PMSF. 80 µg of protein was run on a 5–15% SDS-PAGE gradient gel at 50 V overnight at 4°C and transferred to PVDF membranes by electroblotting overnight at 20 V. Following blocking membranes were incubated with primary polyclonal antibody (rabbit) α-Peg3 at 1/24,000 [67], followed by a 1/5000 poly-clonal anti-rabbit HRP-conjugated secondary antibody (Dako Denmark) and developed using an ECL plus Western Blotting Detection System (Amersham, GE Healthcare).

### DNA extraction

gDNA isolation was carried out by standard organic extraction including an overnight proteinase K treatment at 55°C. DNA was quantified spectrophotometrically and quality was assessed by running 100–500 ng on a 1.5% agarose gel.

### Sex genotyping

The sex of E16.5 embryos was identified by PCR for the sex-chromosome specific genes *Zfy* and *SMCX/Y* using the primers in Table S3.

### Sperm DNA extraction

Two month old male mice were sacrificed and sperm collected from the cauda epididymes and vas deferens as described previously [68], [69]. Extruded sperm and sliced epididymes were suspended in 50 ml of Solution A (0.75 mL 5 M NaCl pH 8; 2.5 ml 0.5 M EDTA; H_2_O to 50 ml) and rocked on a platform for 10 min to release sperm. Non-sperm tissue was removed by 10 minutes of settling, followed by serial centrifugation at 500× g for 15 min, and 700× g for 10 min. Sperm was harvested by centrifugation at 1100× g for 5 min. 200 µl Solution B (0.1 mL Tris-HCl pH 8; 0.2 ml 0.5 M EDTA; 2 ml 10% SDS; 8 ml 100 mM DTT; H_2_O to 10 ml) was added, followed by a standard RNAseA and overnight proteinase K treatment at 55°C. DNA was extracted using DNEasy columns. To accurately quantify sperm DNA concentration Quant-iT PicoGreen was used.

### Sodium bisulphite treatment

Sodium bisulphite mutagenesis was carried out on 1 ug of gDNA per sample using the 2-step conversion protocol of the Sigma Imprint DNA Modification Kit. Two samples with no template were run in parallel to confirm contamination had not occurred during bisulphite treatment.

### Pyrosequencing

Quantification of methylation following bisulphite conversion was carried out by pyrosequencing as previously described [70]. Pyrosequencing primer design was carried out using Qiagen PyroMark Assay Design software 2.0, sequences are given in Table S2. Following PCR, 2.5 µl of each PCR product was run on a gel to ensure specificity of amplification and suitable concentration of product. The biotinylated strand was purified using strepdavidin sepharose high performance beads (GE Healthcare) and PyroMark reagents (Qiagen). Pyrosequencing was carried out on a PyroMark MD pyrosequencer (Biotage) using PyroMark Gold Qp6 SQA reagents (Roche) and quantification of methylated and unmethylated alleles carried out using Pyro Q-CpG 1.0.9 software (Biotage).

## Supporting Information

Figure S1 ROC curve analysis of the microarray data. (A) ROC curves were computed for the imprinted gene set for each array. In order to provide a context for the 4 experimentally-determined (imprinted) ROCs, we also generated ROCs from 100 randomized data sets. The randomized data sets were generated by permuting the gene labels with regard to which genes are imprinted and which are not imprinted. We calculated the area under each of these curves (AUC) and compared each of the 4 experimental data sets to the 100 randomized data sets in terms of this area. F1 E16.5 Liver has an AUC of 0.520232 which is higher than 70 out of 100 randomly labeled sets; F1 E16.5 Placenta has an AUC of 0.53781 which is higher than 91 out of 100 randomly labeled sets. F2 CU Placenta has an AUC of 0.534822 which is higher than 88 out of 100 randomly labeled sets; F2 CU Liver has an AUC of 0.567148 which is higher than 99 out of 100 randomly labeled sets. (B) The distribution of differential expression among the four data sets in terms of false discovery rate.
(PDF)

Figure S2 Imprinted gene list. List of imprinted genes used in the analyses presented in Figure 1 and Figure 3.
(DOC)

Table S1 Quantitative RT-PCR primers. Primer sequences and annealing temperatures of qPCR assays.
(DOC)

Table S2 Pyrosequencing primers. Primer sequences and annealing temperatures of pyrosequencing assays used.
(DOC)

Table S3 Genotyping primers. Primer sequences and annealing temperatures of sex genotyping assays used.
(DOC)
