# Supplementary figures and images for: An Unbiased Assessment of the Role of Imprinted Genes in an Intergenerational Model of Developmental Programming
Source: PLoS Genet. 2012 Apr 12;8(4):e1002605. doi: 10.1371/journal.pgen.1002605 (PMC3325178; doi:10.1371/journal.pgen.1002605)

Figure S1

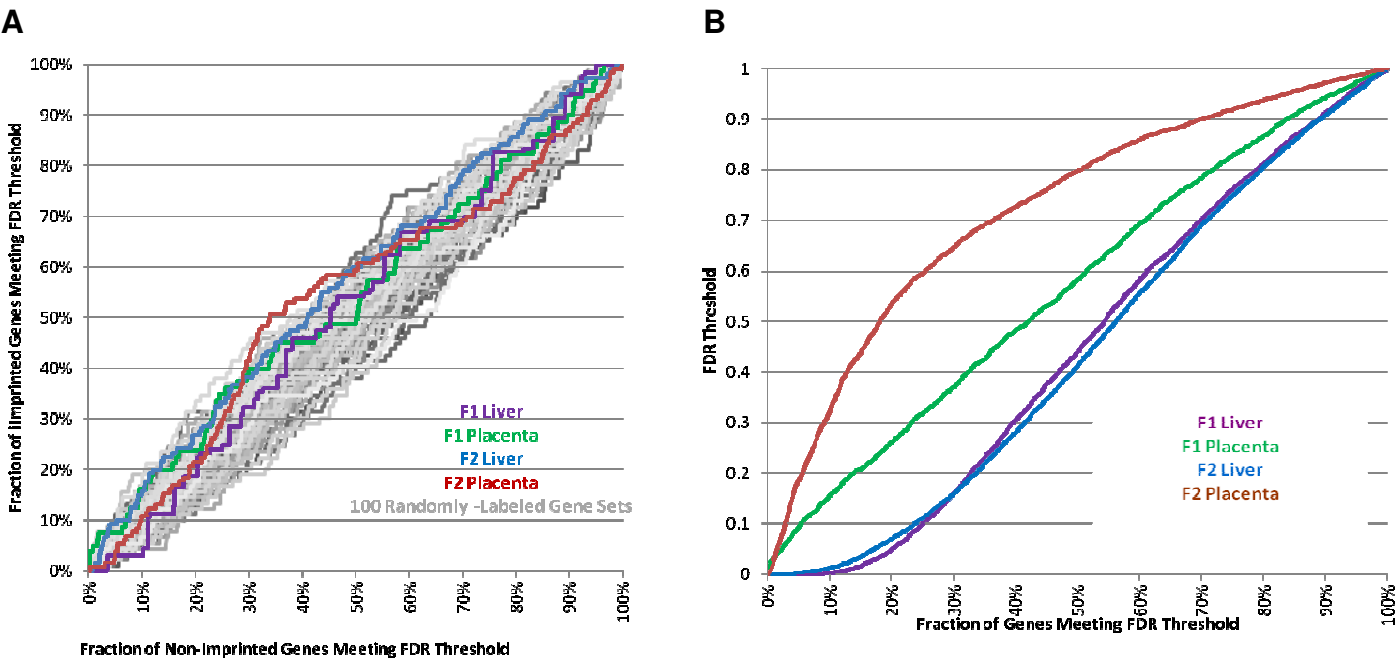

Supplement: Figure S1 — ROC curve analysis of the microarray data. (A) ROC curves were computed for the imprinted gene set for each array. In order to provide a context for the 4 experimentally-determined (imprinted) ROCs, we also generated ROCs from 100 randomized data sets. The randomized data sets were generated by permuting the gene labels with regard to which genes are imprinted and which are not imprinted. We calculated the area under each of these curves (AUC) and compared each of the 4 experimental data sets to the 100 randomized data sets in terms of this area. F1 E16.5 Liver has an AUC of 0.520232 which is higher than 70 out of 100 randomly labeled sets; F1 E16.5 Placenta has an AUC of 0.53781 which is higher than 91 out of 100 randomly labeled sets. F2 CU Placenta has an AUC of 0.534822 which is higher than 88 out of 100 randomly labeled sets; F2 CU Liver has an AUC of 0.567148 which is higher than 99 out of 100 randomly labeled sets. (B) The distribution of differential expression among the four data sets in terms of false discovery rate. (PDF) [file pgen.1002605.s001.pdf]
