# Supplementary material for: An Unbiased Assessment of the Role of Imprinted Genes in an Intergenerational Model of Developmental Programming
Source: PLoS Genet. 2012 Apr 12;8(4):e1002605. doi: 10.1371/journal.pgen.1002605 (PMC3325178; doi:10.1371/journal.pgen.1002605)
Supplement: Figure S2 — Imprinted gene list. List of imprinted genes used in the analyses presented in Figure 1 and Figure 3. (DOC) [file pgen.1002605.s002.doc]

Figure S2

Imprinted gene list

| *A19* | *MEST* |
| --- | --- |
| *Airn* | *Mirg* |
| *Ampd3* | *MKRN3* |
| *Asb4* | *Msuit1* |
| *Ascl2* | *Nap1l5* |
| *Begain1B* | *NDN* |
| *BLCAP* | *NESP55* |
| *Calcr* | *Nespas* |
| *Cd81* | *NNAT* |
| *CDKN1C* | *OSBPL5* |
| *Cntn3* | *Pec2* |
| *Commd1* | *Pec3* |
| *Copg2* | *PEG10* |
| *Copg2as2* | *Peg12* |
| *Ddc* | *Peg13* |
| *Dhcr7* | *PEG3* |
| *DIO3* | *PHLDA2* |
| *DLK1* | *PLAGL1* |
| *Gatm* | *PPP1R9A* |
| *GNAS_EXON1A* | *Rasgrf1* |
| *GNASL* | *Rtl1* |
| *GNASXL* | *Scin* |
| *Gpr1* | *Sfmbt2* |
| *Grb10* | *SGCE* |
| *GRB10_major* | *SLC22A18* |
| *Gtl2* | *Slc22a2* |
| *H13* | *Slc22a3* |
| *H19* | *Slc38a4* |
| *Htr2a* | *SNRPN* |
| *IGF2* | *Tfpi2* |
| *IGF2AS* | *Th* |
| *Igf2r* | *Tnfrsf23* |
| *Impact* | *Tssc4* |
| *INPP5F_V2* | *UBE3A* |
| *Ins2* | *Usp29* |
| *KCNK9* | *Wt1* |
| *KCNQ1* | *Zdbf2* |
| *KCNQ1OT1* | *Zfp264* |
| *KLF14* | *Zim1* |
| *MAGEL2* | *ZIM2* |
| *MCTS2* | *Zim3* |
| *Meg8* | *Zrsr1* |
