# Supplementary material for: An Unbiased Assessment of the Role of Imprinted Genes in an Intergenerational Model of Developmental Programming
Source: PLoS Genet. 2012 Apr 12;8(4):e1002605. doi: 10.1371/journal.pgen.1002605 (PMC3325178; doi:10.1371/journal.pgen.1002605)
Supplement: Table S1 — Quantitative RT-PCR primers. Primer sequences and annealing temperatures of qPCR assays. (DOC) [file pgen.1002605.s003.doc]

### Table S1: Quantitative RT-PCR primers

| Transcript | Primers | Annealing temp °C | Reference (if applicable) |
| --- | --- | --- | --- |
| *HPRT* | F: CAGGCCAGACTTTGTTGGAT  R: TTGCGCTCATCTTAGGCTTT | 60 |  |
| *Dlk1* | F: GAAAGGACTGCCAGCACAAG  R: CACAGAAGTTGCCTGAGAAGC | 60 |  |
| *Gtl2* | F: GGACACACGGACACAGACA  R: TGTCCCACAGGAAATGTGCAA | 60 |  |
| *Igf2 (total)* | F: CGCTTCAGTTTGTCTGTTCG  R: GGGGTGGCACAGTATGTCTC | 60 |  |
| *Igf2P0* | F: GCATCCCCGGTCCTCTTTAT  R: GCTCTGGCTGGACGAGAAGT | 60 | [70] |
| *H19* | F: TACCTGCCTCAGGAATCTGC  R: GTTGGCCATGAAGATGGATT | 60 |  |
| *Igf2r* | F:GCCTTCAGATTCACAGCACA  R: TCATGCTTCTGTAACTTGTCATCA | 60 |  |
| *Grb10 (total)* | F: TGCACCACTTCTTGAGGATG  R: ACCAGTGAGCTCCGGAAATG | 60 | [33] |
| *Grb10 maternal-type isoform* | F: GAGCACGAAGTTTCCGCGCA  R:CTGGTTGGCTTCTTTGTTGTGG | 60 | [33] |
| *Grb10 paternal-type isoform* | F: CAAGGTACAGAGCTAGGACG  R: CTGGTTGGCTTCTTTGTTGTGG  100nM | 50 | [33] |
| *Zac1* | F: TTCGTCACCCTGGAGAAGTT  R: GGTCTGGAGGTGGTTCTTCA | 60 |  |
| *Snrpn* | F: AGGTCGAGGTCCAGGTCAA  R: AATCCACCACAGGAACTTGC | 65 |  |
| *Peg10* | F: GTGGCATCGCAGAGGAAT  R: GTGAGAGGGGCTTCACTCC | 65 |  |
| *Peg3* | F: ACTCACCACTCCGTTGGAGAGTTT  R: TTTCTCTCCCACTTCGGCTCATGT | 60 |  |
| *Slc38a4* | F: GCTTCTTCTGCCACTATGCTGA  R: TCCAGTCACAAGGAAGACCAAA | 60 | [70] |
| *Phlda2/IPL* | F: GCGCTCTGAGTCTGAAATGC  R: CTCCTGGGCTCCTGTCTGAT | 60 |  |
| *Cdkn1c* | F: GAAGGACCAGCCTCTCTCG  R: ACGTTTGGAGAGGGACACC | 63 |  |

All primers were used at 400nM unless otherwise specified.
