# Supplementary material for: An Unbiased Assessment of the Role of Imprinted Genes in an Intergenerational Model of Developmental Programming
Source: PLoS Genet. 2012 Apr 12;8(4):e1002605. doi: 10.1371/journal.pgen.1002605 (PMC3325178; doi:10.1371/journal.pgen.1002605)
Supplement: Table S2 — Pyrosequencing primers. Primer sequences and annealing temperatures of pyrosequencing assays used. (DOC) [file pgen.1002605.s004.doc]

### Table S2: Pyrosequencing primers

| Gene | Primers | Annealing temp °C |
| --- | --- | --- |
| *Peg3 DMR* | F: TTGGATTGGTTAGAGAGGAAGT  R: [biotin] ACAATCTAATACACCCACACTAA  Seq: GGAGAGATGTTTATTTTG | 57 |
| *Snrpn DMR* | F: TTGGTAGTTGTTTTTTGGTAGGAT  R: [biotin]TCCACAAACCCAACTAACCTTC  Seq GTGTAGTTATTGTTTGGGA | 54 |
| *Dlk1/Dio3 IgDMR* | F: GTGGTTTGTTATGGGTAAGTTT  R: [biotin] CCCTTCCCTCACTCCAAAAATTAA  Seq TGGTTTATTGTATATAATGT | 54 |
| *H19 DMR* | F: GGGGGGTAGGATATATGTATTTTT  R:[biotin]ACCTCATAAAACCCATAACTATAAAATCAT  Seq GTGTGTAAAGATTAGGG | 54 |
